# Multi-ignition fire complexes drive extreme fire years and impacts

**DOI:** 10.1126/sciadv.adx6477

**Published:** 2026-01-02

**Authors:** Rebecca C. Scholten, Tirtha Banerjee, Yang Chen, Andrea Delgado, Ajinkya Desai, Ziming Ke, Tianjia Liu, Douglas C. Morton, David A. Peterson, Qi Tang, Sander Veraverbeke, Jishi Zhang, James T. Randerson

**Affiliations:** ^1^ Department of Earth System Science, University of California, Irvine, CA 92697, USA.; ^2^ Department of Civil and Environmental Engineering, University of California, Irvine 92697, CA, USA.; ^3^ Lawrence Livermore National Laboratory, Livermore, CA 94550, USA.; ^4^ Division of Atmospheric Science, Desert Research Institute, Reno, NV 89512, USA.; ^5^ Department of Geography, University of British Columbia, Vancouver, BC, Canada.; ^6^ NASA Goddard Space Flight Center, Greenbelt, MD 20771, USA.; ^7^ University of Maryland, College Park, MD 20742, USA.; ^8^ US Naval Research Laboratory, Monterey, CA 93943, USA.; ^9^ Faculty of Sciences, Vrije Universiteit Amsterdam, Amsterdam, Netherlands.; ^10^ School of Environmental Sciences, University of East Anglia, Norwich, UK.

## Abstract

Climate change is intensifying fire behavior, with the largest and fastest-spreading fires causing the greatest impacts on people and ecosystems. Yet the mechanisms driving variability and trends in large fires remain poorly understood. Using 12-hour satellite-derived fire tracking data from 2012 to 2023, we show that the merging of separate ignitions into multi-ignition complexes is a key process amplifying fire size and destructive potential across temperate and boreal ecoregions. Multi-ignition fires account for 31% of the burned area in California and 59% in the Arctic-boreal domain, spread faster and persist longer than single-ignition fires, and disproportionately contribute to extreme fire years in California, Canada, and Siberia. They also generate stronger atmospheric feedbacks, produce more pyrocumulonimbus events, and strain firefighting capacity by dispersing suppression resources. Recognizing and accounting for fire-merging dynamics are critical for improving wildfire prediction, risk assessment, and management.

## Teaser

When lightning ignites multiple fires that merge, the resulting complexes spread faster and cause greater destruction.

## INTRODUCTION

The past decade has seen increasing wildfire disasters contributing to economic losses and ecosystem damage (*1*–*7*). Climate warming has been linked to increasing burned areas, especially in forested regions across global biomes (*8*–*11*). The devastating impacts from wildfires are, however, often caused by extreme fire behavior, spread rate, and size (*12*–*14*), and trends in such fire extremes are poorly understood. New climate extremes may facilitate fires that escape initial control (*15*) or synchronize fire behavior within and across regions due to persistent large-scale weather patterns (*16*–*19*), requiring triaging of fire suppression resources.

Globally, fire sizes follow a log-normal distribution, with the vast majority of fires remaining small (*20*–*22*). Large fire seasons are often dominated by a few rare, large events (*23*). Fires usually originate from a single anthropogenic or natural point of ignition. In contrast, multi-ignition fires are wildfires that result from multiple separate ignition points that eventually merge into a single fire perimeter. Although there are prominent examples of extremely large and destructive multi-ignition fires in regions such as California in 2020 and Canada in 2023, there has been little quantitative assessment of these events or of how their impacts differ from those of single-ignition wildfires.

Here, we assessed the contribution of multi-ignition fires to regional burned area and loss and damage in temperate and boreal forest ecosystems using satellite data from 2012 to 2023. We examine their causes, impacts, and the atmospheric and human system feedbacks that may amplify their destructiveness. Our analysis leverages recent advances in subdaily fire tracking, drawing on perimeter data from the California Fire Events Data Suite (FEDS version 2) and the Arctic-boreal Fire Atlas (ABFA) (*24*, *25*). Together, these datasets provide the most spatially extensive observations now available for tracking fire dynamics at subdaily time steps across diverse fire regimes, enabling us to identify and characterize multi-ignition fires as a broad-scale phenomenon. The datasets track individual large (>4 km^2^) fire events’ starting location, growth, and merging. Each fire perimeter at a 12-hour time step is derived from active fire observations from the Visible Infrared Imaging Radiometer Suite (VIIRS) instrument on the Suomi National Polar-orbiting Partnership satellite, with a nadir spatial resolution of ~375 m. Fire perimeters were validated using official records in regions for which these were available (California, Alaska, and Canada). We classified fire events as multi-ignition fires if the fire tracking algorithms identified multiple nearby fire starts that remained independent for two or more time steps before physically merging (see Materials and Methods). We note that this definition differs from the term “complex fire” in fire management contexts, which refers to fires managed by a single Incident Management Team that shares resources and equipment. The number and location of ignition points for the fires we identified as multi-ignition fires agreed reasonably well with state and federal records in California and Canada (table S1 and fig. S1).

## RESULTS

### Disproportionate contribution to burned area and impacts

**Fig. 1.**
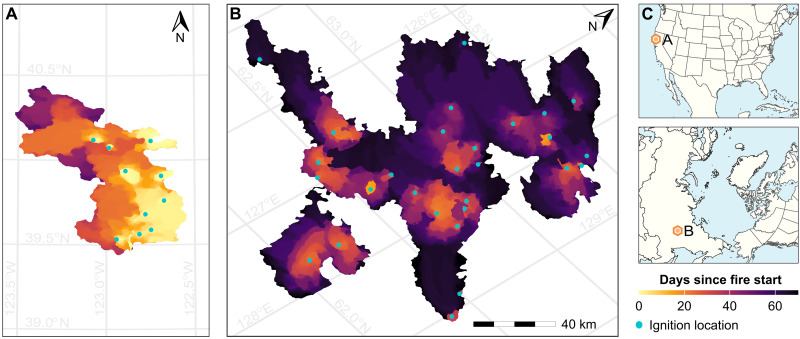
Since 2012, the largest fires in California and the Arctic-boreal region began as multiple ignitions that later merged. (**A**) The August Complex fire in northern California in 2020 had 10 separate ignition points (denoted by turquoise dots). (**B**) A wildfire in Yakutia, Russia, in 2021 had 27 ignition points. The maps illustrate the fire starts from FEDS/ABFA fire tracking datasets along with the 12-hourly progression of these fires (in color), showing only fire perimeters up to the 99th percentile of their final area. See fig. S1 for a comparison of fire tracking fire starts with official governmental records for the August Complex fire and a fire in Canada. Both maps are on the same spatial and temporal scale. (**C**) The location of these two fires in California and Siberia.

**Fig. 2.**
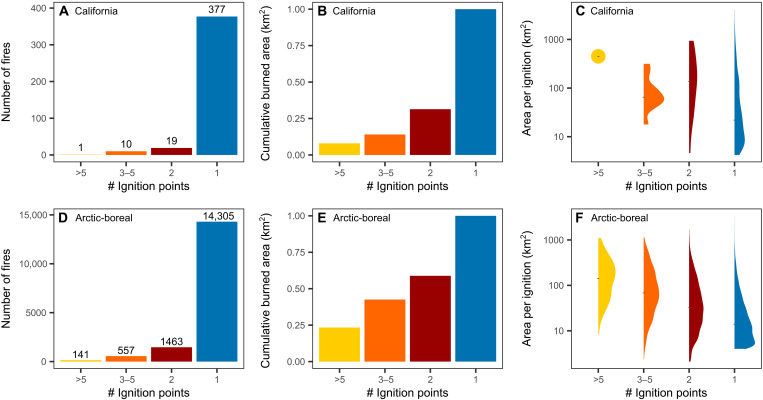
Although they are relatively rare, multi-ignition fires have a disproportionate effect on burned areas in California and Arctic-boreal regions. (**A** and **D**) The number of fires aggregated by the number of fire starts from the FEDS and ABFA fire tracking datasets. (**B** and **E**) Cumulative burned area as a function of the number of individual fire starts in a fire. (**C** and **F**) The area burned by each individual ignition in single- and multi-ignition fires, on a log scale.

The largest individual fires between 2012 and 2023 in California and the Arctic-boreal domain were multi-ignition fires (Fig. 1). The largest fire on record in California, the August Complex fire, burned 4489 km^2^ in 2020 and had 10 fire starts. Five of the 10 largest fires in California during 2012–2023 were multi-ignition fires. In Yakutia, Eastern Siberia, 27 fire starts merged in 2021 to ultimately burn a total area of 15,759 km^2^. Multi-ignition fires accounted for 7% of the total number of fires larger than 4 km^2^ in California and 13% in the Arctic-boreal domain (Fig. 2, A and D). However, they contributed to 31% of the burned area in California and 59% of the burned area in the Arctic-boreal domain since 2012 (Fig. 2, B and E). Normalizing by the number of separate ignition points, multi-ignition fires burned more area than single ignition fires (Fig. 2, C and F) in both California (median: 103.2 km^2^ versus 22.0 km^2^, *P* < 0.001; unless otherwise noted, all *P* values are from a Mann-Whitney test) and the Arctic-boreal domain (median: 44.4 km^2^ versus 14.0 km^2^, *P* < 0.001; table S2). These fires occurred more frequently in California’s northern coastal and mountain regions (fig. S2A) and were widely distributed across North America and Siberia in the Arctic-boreal domain (fig. S2B).

**Fig. 3.**
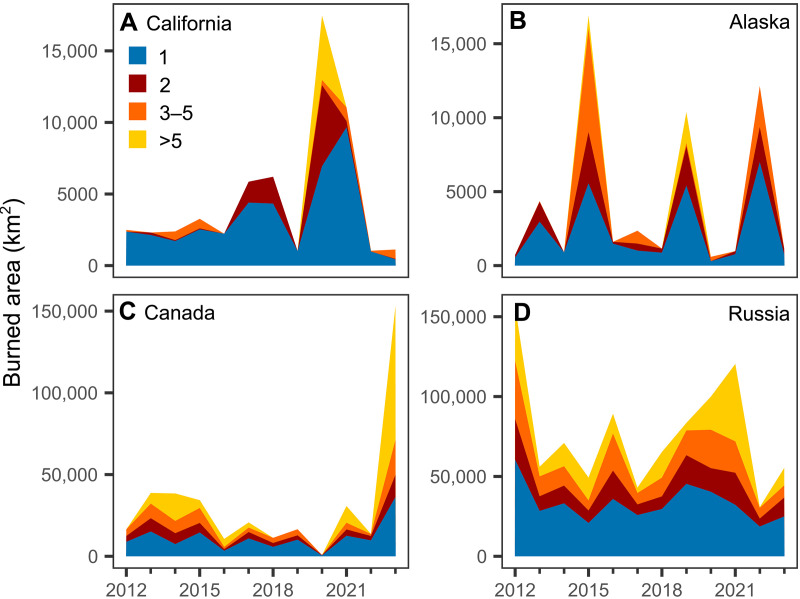
Multi-ignition fires have a disproportionate effect on interannual variability in burned area and extreme fire years. (**A**) California, (**B**) Alaska, (**C**) Canada, and (**D**) Russia. Colors represent the burned area contributed by fires with a given number of ignitions. Average percentages of burned area from multi- and single-ignition fires for average and large fire years are shown in table S3.

Multi-ignition fires strongly influenced the interannual variability in burned area in temperate and boreal regions (Fig. 3 and table S3). The coefficient of variation (CV) of burned area associated with multi-ignition fires was higher than the CV of burned area associated with single-ignition fires in all regions. Notably, large fire years in all regions consistently had larger contributions from multi-ignition fires than small fire years (Fig. 3 and table S3). For example, multi-ignition fires accounted for 42% of the burned area in California’s largest fire years (2020 and 2021), compared to only 21% for all other fire years since 2012. Likewise, 76% of the burned area in Canada’s extreme 2023 fire season originated from multi-ignition fires. In Russia, multi-ignition fires contributed to 67% of the burned area in high-fire years during 2012 and 2021.

**Fig. 4.**
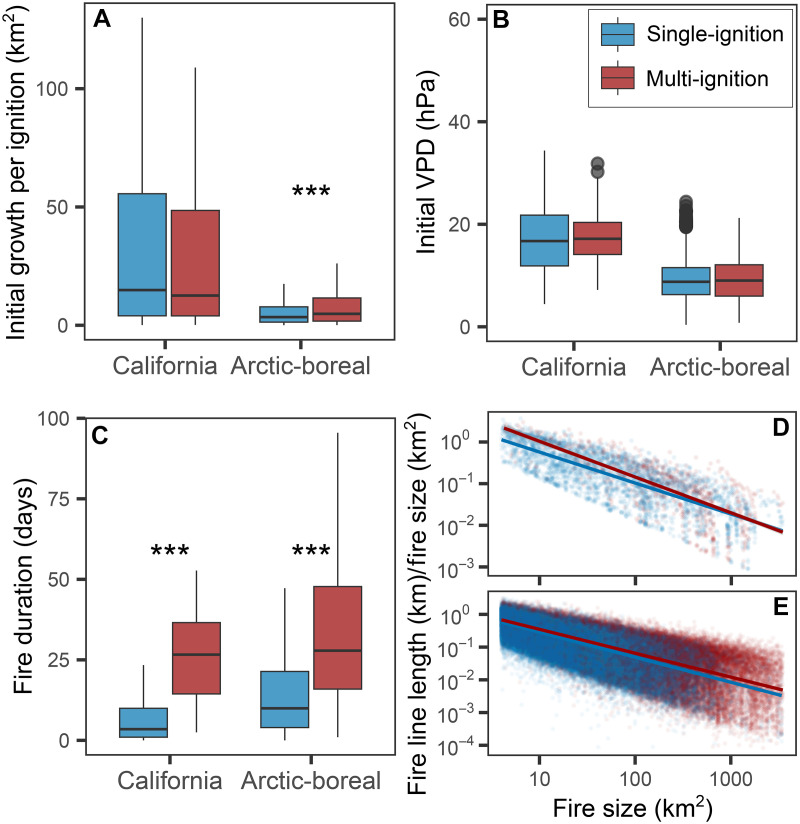
Mechanisms for the disproportionate size of multi-ignition fires. (**A**) Initial growth, estimated as the fire size on day 4 normalized by the number of initial ignitions, for single- and multi-ignition fires. (**B**) Initial VPD at ignition locations averaged over the first 4 days after ignition. (**C**) Fire duration (corrected for residual smoldering by computing the date and time when 99% of the final fire size was reached). Boxplots (A) and (C) only include the interquartile range for better visibility since the data are strongly skewed. Asterisks indicate significance level (***: 0.001) for statistical testing for group differences [Mann-Whitney test for (A) and (C), *t* test for (B)]. (**D** and **E**) Ratio of fire line length to fire size for 12-hourly time steps of all fire perimeters larger than 4 km^2^ in California (D) and the Arctic-boreal domain (E). Only perimeters up to 99% of the final fire size of each fire are retained. Model intercepts and slopes are significantly different between single- and multi-ignition fires for both regions (ANOVA *P* < 0.05). Information on statistical tests and sample sizes are found in table S2.

Recent fire perimeters can restrict the growth of a newly occurring fire by reducing the amount of available fuel (*26*). This limitation from fuel availability would be expected to constrain the spread and size of multi-ignition fires relative to more widely spaced fires with the same number of ignition points. However, our results indicate that fires grow larger when burning in a multi-ignition event. This counterintuitive behavior may be related to fire behavior traits that enable faster initial growth and longer duration of these fires. Multi-ignition fires lasted longer on average than single ignition fires in California (median: 26.8 days versus 3.5 days, *P* < 0.001) and in the Arctic-boreal domain (median: 28.0 days versus 10.0 days, *P* < 0.001; Fig. 4 and table S2). Four days after ignition, multi-ignition fires burned more area per fire start compared to single-ignition fires in the Arctic-boreal domain (median: 8.4 km^2^ versus 5.7 km^2^, *P* < 0.001; table S2), but not in California. This faster initial growth could not be explained by more favorable weather conditions. For instance, vapor pressure deficit (VPD) values during the first 4 days of multi-ignition fires were similar compared to those observed for single-ignition fires in both regions (Fig. 4B). Multi-ignition fires may exhibit enhanced spread and persistence due to factors such as a longer active fire line relative to the fire area compared to single-ignition fires [Fig. 4, D and E; analysis of variance (ANOVA) test, *P* < 0.001] and potential fire interaction effects (*27*).

We used ICS-209 Incident Status Summary reports to assess the fire management resources and damages associated with fires in California. ICS-209 reports are standardized incident status summary forms used by US wildfire management agencies to document and communicate key fire characteristics, resources deployed, and suppression progress during active wildfire incidents. Multi-ignition fires in California were significantly more expensive to manage (median cost: $51 million dollars) and required higher levels of fire suppression resources than single-ignition fires (median cost: $12 million dollars, *P* < 0.001; table S4). Multi-ignition fires also required twice the number of personnel on average (*P* < 0001; table S4). When normalizing resources by the number of ignitions for each fire, multi-ignition fires were still more costly than single-ignition fires by a factor of 2, despite lower or comparable deployment of fire suppression resources, including personnel (table S4). Differences were even more pronounced regarding damages, with multi-ignition fires leading to about three times more threatened structures and evacuees per ignition as well as significantly more destroyed structures and affected civilians per ignition compared to single-ignition fires (table S4). Multi-ignition fires also disproportionally affected the health of firefighting personnel (*P* = 0.04), indicating the potential for elevated hazards from more extreme behavior or resource limitation. ICS-209 reports from multi-ignition fires document potential underlying causes of the elevated risks for firefighters, including understaffing, extended mobilization periods, and shorter resting periods due to longer shifts (table S5). Limited data on threatened and damaged structures per fire were also available from the Alaska Wildland Fire Maps (AWFM) Fire Location dataset. In Alaska, multi-ignition fires resulted in more damage to structures per ignition than single-ignition fires (*P* = 0.03). In contrast, the number of threatened structures did not differ significantly. Overall, about 9.9% of multi-ignition fires in Alaska resulted in structure damage compared to 3.8% for single-ignition fires.

### Dry lightning causes clustered fire ignitions

In California, the probability of individual fires merging was 16 ± 12% during 2012–2023 (table S6). We simulated the merging rate of fires in California expected from random chance by spatially distributing the observed number of fire ignitions in a given year using an observed fire probability map that accounted for lower burn probabilities within recent fire perimeters, as a consequence of decreases in available fuels. We then assessed the merging probability by comparing the distances between simulated ignitions with radii derived from observed fire sizes from the same year. We found that simulated merging probabilities were consistently lower by several orders of magnitude than the observed merging rate (table S6). An essential prerequisite of multi-ignition fires is thus the spatiotemporal clustering of ignitions in a region with sufficient fuel continuity to enable fire spread. The median distance between ignitions within a multi-ignition fire was 9.5 km in California and 8.2 km in Arctic-boreal regions (table S2). Multiple simultaneous ignition points separated by distances on this length scale (~5 to 13 km) appear optimal for creating large multi-ignition fire events.

**Fig. 5.**
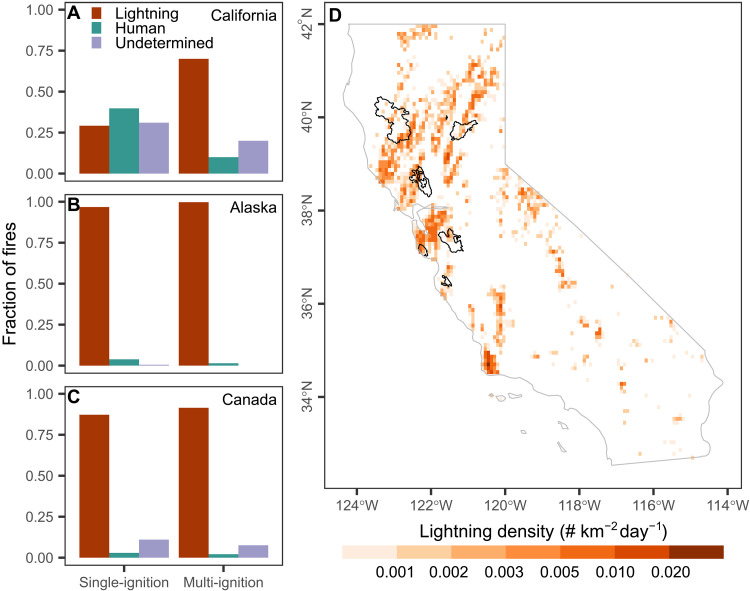
Lightning causes most of the multi-ignition fires in California and Arctic-boreal regions. (**A** to **C**) Fraction of ignitions of single- and multi-ignition fires caused by lightning, anthropogenic, and undetermined sources. (A) California (377 single-ignition and 30 multi-ignition fires), (B) Alaska (369 single-ignition and 71 multi-ignition fires), (C) Canada (2214 single-ignition and 334 multi-ignition fires). Fire causes were assessed by overlaying large FEDS perimeters (>4 km^2^) with Fire and Resource Assessment Program (FRAP) fire perimeters (for California), the National Burned Area Composite (NBAC; for Canada), and the AWFM Fire Location data (for Alaska). (**D**) Monthly lightning density from the World Wide Lightning Location Network (*62*) in California in August 2020 shows thunderstorm tracks over Northern California and corresponding perimeters of multi-ignition fires ignited by this event over the period from 16 to 19 August.

Lightning strikes are a primary cause of clustered ignitions. Data on ignition sources for California fires revealed that multi-ignition fires are predominantly ignited by lightning (Fig. 5A). This is in contrast to most fires in the region, which are ignited by humans (*28*). In central and northern California, moisture plumes linked to the remnants of tropical cyclones and the summertime North American monsoon circulation can support widespread thunderstorm development that produces clustered lightning strikes (*29*). Thunderstorms in these regions often develop over a layer of dry air near the ground, which results in high cloud bases and rapid evaporation of precipitation before it reaches the ground (*30*). Hundreds of fire ignitions have been observed within a few days after these high-based, “dry thunderstorm” outbreaks that can quickly overwhelm initial fire attack capabilities (*31*). One of the most notable events occurred in August 2020, when such storms sparked five of the six largest fires of the season over a 4-day period (Fig. 5D).

Arctic-boreal regions are more prone to summer thunderstorm activity, and lightning is a more critical ignition source compared to wildfires in California. Large-scale regional patterns of fuel moisture, shaped by summer drought, are essential for determining ignition efficiency in these regions (*32*, *33*) and can promote clusters of ignitions that are prone to merging. According to government fire records, lightning was responsible for all but one multi-ignition fire in Alaska and 98% of those in Canada (Fig. 5, B and C).

#### Multi-ignition fires can overwhelm firefighting efforts

When multiple fires ignite simultaneously across a region, available firefighting resources can be quickly overwhelmed, requiring triage—that is, prioritizing which fires or management objectives to address first. Fighting fires on multiple fire fronts also requires more complex management structures and overhead. This strain can create a self-reinforcing feedback that allows fires to grow larger and increase both, the damage they cause and the hazards faced by firefighters. Insufficient suppression resources, including crews, overhead personnel, aircraft, and engines, are frequently described in ICS-209 reports for multi-ignition fires in California (table S5). Reports often note such limitations, especially in the initial days after ignition, which present a crucial window for initial attack and cost-efficient containment.

Many of the most prominent and destructive examples of multi-ignition fires in California happened in large fire years, when resources were divided across multiple simultaneously burning fires. However, analysis of initial resource allocation (up to 4 days after ignition) revealed that while large fire seasons significantly reduced resources available to manage single-ignition fires, multi-ignition fires consistently received less personnel per ignition regardless of fire season severity (fig. S3 and table S7). Differences in the allocation of engines and equipment between single- and multi-ignition fires were largest in average fire seasons. This disparity may partly reflect that lightning-caused fires often occur in remote locations where resource allocation faces greater logistical challenges and cost-benefit constraints. Furthermore, large lightning events such as the August 2020 lightning storm can deliver hundreds of fire starts within 1 or 2 days, creating acute resource shortages when a substantial fraction of a season’s fires ignite simultaneously.

ICS-209 reports further document that multi-ignition fires display aggressive fire behavior, including substantial runs due to the merging of fires or simultaneous crown fire initiations and spotting, which elevates hazards and challenges to containment. Sharing of resources such as aircraft is described as leading to difficulties in assessing fire size and growth, hampering suppression planning. Other specific challenges for the suppression of multi-ignition fires are a scarcity of specialized crews and extended deployments, which lead to prioritization of point protection and fires being left understaffed. While event-specific data on resource allocation were not available for Arctic-boreal regions, considerable resource strain has also been reported, for example, in Canada in 2023, where multi-ignition fires contributed to 76% of the area burned (*34*).

**Fig. 6.**
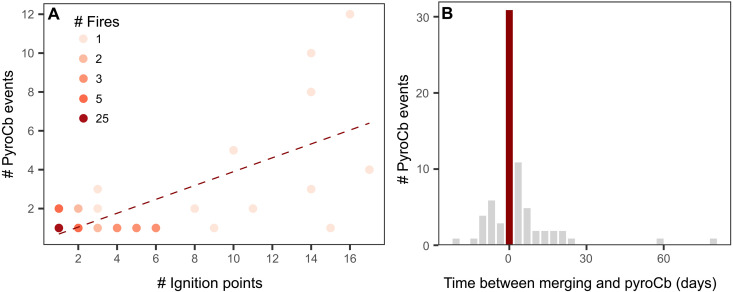
In Arctic-boreal regions, multi-ignition fires are associated with more pyroCb events than single-ignition fires. (**A**) Scatterplot of the number of pyroCb events caused by a fire and the number of ignitions for that fire (Pearson’s *r* = 0.68, *P* < 0.001, Spearman’s rho = 0.41, *P* = 0.001). Data include 60 single- and multi-ignition fires with 108 associated pyroCb events in 2023. The color of the points represents the number of fires in each bin. (**B**) Minimum time between a merging event and a pyroCb in multi-ignition fires. The dataset in (B) includes 30 multi-ignition fires with 73 associated pyroCbs.

Multi-ignition fires are also more likely than single-ignition fires to generate extreme, plume-driven fire behavior and pyroconvection, which can culminate in fire-triggered thunderstorms, known as pyrocumulonimbus events (pyroCbs). On the basis of a global inventory of pyroCb occurrences (*35*–*37*), we found that 67% of pyroCbs in Russia and Canada in 2023 were associated with multi-ignition fires. The number of pyroCbs generated by a fire was linked to the number of separate ignition points in Canada and Russia in 2023 (Pearson’s *r* = 0.68, *P* < 0.001; Fig. 6A). The merging of two fire lines can increase the surface area with high sensible heat fluxes needed to trigger deep convection (*38*, *39*) and create a rotating column of rising air which can induce pyroconvection (*40*). Notably, 53% of the 73 pyroCbs observed over multi-ignition fires in Canada and Russia in 2023 occurred between 1 day before and 3 days after a merging event, with 22% occurring on the day of merging (Fig. 6B). In contrast, if pyroCbs were to have occurred randomly over the lifetime of multi-ignition fires, the expected probability of occurring on the day of a merging event would be only 1% (*z*-score = 17.5, *P* < 0.001).

In California, only 19 pyroCbs linked to 10 FEDS fires were identified between 2012 and 2023, providing too few cases for robust statistical analysis. Multi-ignition fires had a slightly higher likelihood of producing pyroCbs (6.6% versus 4.5% for single-ignition fires), but only two pyroCb events occurred in fires that met our multi-ignition definition. This indicates that factors other than fire merging—particularly explosive fire growth—can drive pyroCb formation, as many pyroCb-producing fires were extremely large regardless of ignition type or number.

PyroCbs also reduce suppression efficiency by inducing unpredictable and hazardous extreme fire behavior (*41*, *42*). Lightning strikes produced by pyroCb activity (a unique type of high-based dry thunderstorm) (*43*) can limit aircraft deployment, hinder airborne suppression efficiency (*44*), and ignite additional spot fires tens of kilometers away from the fire front (*45*–*47*). Plume-driven fires generate irregular wind patterns, which may push fire fronts and spot fires in unexpected directions, jeopardizing containment lines (*36*) and elevating risks for firefighters and communities (*48*). Physical interactions between fires burning in close vicinity can further contribute to the large size of multi-ignition fires. For example, fire merging can be accelerated when large fires act as attractors for smaller fires (*49*) through changes in surface winds that develop in response to the intense surface heating and deep convection generated by the larger fire. Entrainment effects are ubiquitous even at meter scales (*50*) and may be most far-reaching for plume-driven fires that generate pyroCbs (*38*, *39*). In a simulation of the Dixie Fire in California—which produced a pyroCb on 9 August 2021, while exhibiting two distinct fire fronts—chaotic and highly heterogeneous wind patterns developed (fig. S4). Downbursts from pyroCbs can lead to extreme fire spread episodes, which can further accelerate merging of fires (*48*). Interactions between physical factors induced by fire attraction, extreme fire behavior, and reduced suppression efficiency due to increased firefighting hazards and resource strain are likely to exacerbate the impacts of multi-ignition fires in a positive feedback loop, as shown in fig. S5.

## DISCUSSION

Tropical and mid-latitude storm systems can facilitate thunderstorm development and associated fire ignitions from lightning strikes over large areas, when they coincide with a dry airmass near the surface during or following a heatwave. The risk of dry lightning strikes across temperate and boreal regions, as well as future trends in such events, is not now well understood. Increasing regional summer heat waves and droughts, along with associated increases in fire weather (*11*), heighten the chances of thunderstorms delivering dry lightning because intense surface heating can evaporate rain before it reaches the ground (*29*). For example, modeling studies in Arctic-boreal regions suggest that the number of fires escaping initial attack is rising (*31*) due to increases in lightning ignitions (*32*, *51*). Understanding the compound risk of persistent heatwaves and high-based thunderstorms across regions is therefore crucial to better forecast the occurrence of multi-ignition fires.

The identification and analysis of multi-ignition fires have only become possible because of recent advances in fire tracking, and uncertainties associated with these data products are considerable due to the temporal and spatial limitations of medium-resolution satellite observations. The impact of multi-ignition fires described here is likely underestimated, and our estimate of the number of multi-ignition fires is likely conservative since our approach cannot disentangle multiple ignitions that burn together very quickly. Furthermore, our dataset does not represent fast-moving grass fires well due to the limited temporal resolution of the satellite imagery. Dense smoke and clouds, for example, from pyrocumulonimbus formations from large fires, can obscure satellite detection and may limit our ability to identify and differentiate between new ignitions and spot fires. As shown in table S8 using hourly fire observations from the Geostationary Operational Environmental Satellites (GOES) instrument, solely increasing the temporal resolution of satellite active fire detections does not improve the ability to distinguish separate ignitions. Instead, higher-resolution satellite imagery of thermal anomalies of fire in both time and space is critically needed to better understand the dynamics of multi-ignition fires and their impacts. Targeted field measurements of fire behavior are also essential for an improved understanding of the processes and feedbacks associated with multi-ignition fires and their effects on atmospheric dynamics and composition.

Multi-ignition fires can become more destructive due to feedback between physical processes and human systems (fig. S5). Given their association with more extreme fire behavior, we hypothesize that they may also generate disproportionate impacts through mechanisms such as elevated fire severity. An essential next step in this context is to quantify tree mortality and other measures of fire severity for multi-ignition and single-ignition fires, for example, using remotely sensed proxies, to explore whether the observed differences in fire behavior described here yield long-term ecosystem impacts. Furthermore, new management approaches may be needed to address these fires’ unique characteristics. Early identification and improved assessment of the risks posed by dry lightning storms for generating synchronized ignitions during hot summer periods may enable more effective predeployment of fire suppression resources to vulnerable areas. At the same time, honing strategies to optimize fire suppression resources among multiple clustered fires, especially during periods of resource strain, seems critical for limiting multi-ignition fire growth and damages. Identifying the fire behavior components that make these fires particularly hazardous and difficult to suppress can support resource allocation decisions and help prevent stress from triaging.

## MATERIALS AND METHODS

### Fire tracking data

We used satellite-derived fire tracking data for 2012–2023 from the California FEDS and ABFA. Both datasets, which contain 12-hourly fire perimeters for all large fire events, were produced using VIIRS active fire detections and an improved version (version 2) of the fire tracking FEDS algorithm. For both California and the Arctic-boreal domain, regional optimization was performed to calibrate land cover–specific spatial and temporal thresholds, determining whether a new fire cluster belongs to an existing fire or constitutes a new fire start (*24*, *25*). The proximity threshold for assigning a cluster to an existing fire was optimized to account for fuel type–specific variations in spread rates and ranged from 1 to 5 km to cover gaps in fire progression induced by observation gaps due to the 12-hourly overpasses (*24*, *25*). The fire tracking algorithm also enables the merging of two existing, actively expanding fires using the same spatial proximity thresholds. A merging event occurs when a new cluster of fire detections is recorded between two actively expanding fires so that the new fire locations are within the attribution distance threshold of both fires. Actively expanding fires were defined as fires that had new detections within a land cover– and biome-specific temporal threshold (up to 5 days for FEDS and up to 30 days for ABFA), which was based on typical smoldering times and validated for each dataset (*24*, *25*). Multi-ignition fires were identified as having at least two fire starts that either fully merged or burned simultaneously within the land cover–specific distance threshold, indicating a high likelihood of merging between overpasses.

Following the minimum mapping unit of the widely used Monitoring Trends in Burn Severity Program (MTBS) for the western United States, we filtered all final perimeters by size (1000 acres or 4.05 km^2^). Fires greater than 4 km^2^ account for 79% of the burned area in California and 98% in the Arctic-boreal domain. Since agricultural fires in California are generally small, they were automatically excluded by the size filtering criteria. For the Arctic-boreal domain, we further removed agricultural fires following the filtering routine described by Scholten *et al.* (*25*). These constraints yielded a set of 830 fires in California and 16,775 fires in the Arctic-boreal domain for the period from 2012 through 2023.

For California, we further removed all fire polygons that did not coincide in space and time with a Fire and Resource Assessment Program (FRAP) or MTBS fire perimeter for California of the same year. We restricted our analysis to this smaller set of known fires to optimize the number of ignitions and to extract data on firefighting resource use and impacts, which are not available for all fires in the FEDS database. FRAP is the most complete digital record of fire perimeters in California, but it is still incomplete. Specifically, several large fires within Camp Pendleton, Fort Hunter Liggett, Camp Roberts, and other military bases, as well as along the border with Mexico, were missing in FRAP. We therefore used MTBS perimeters for these missing fires. FRAP recorded a total of 515 fires in California in 2012–2023, and MTBS recorded an additional 41 fires. We matched fires based on spatial and temporal overlap. For the temporal overlap, we matched the “ALARM_DATE” attribute of FRAP or the “Ig_Date” attribute for MTBS perimeters, with the start time derived from FEDS, allowing for a 10-day offset in both directions to account for temporal uncertainties in all three datasets. The crosswalk removed small and recurring anthropogenic fires in the Central Valley and the Sierra Nevada foothill regions and returned 407 unique fires.

### Identification of fire starts

Both datasets, FEDS and ABFA, record all fire start locations for each fire throughout its lifetime as detected by the VIIRS sensor. The original fire tracking system, therefore, does not inherently differentiate between actual wildfire ignitions and other features that resemble fire starts, including spot fires, back-burns ignited for fire management purposes, or artifacts created by detection gaps (for example, because of dense clouds or smoke). To optimize our algorithm parameters and accurately identify wildfire ignitions, we used reference ignition location data for California from the Fire Program Analysis Fire Occurrence Dataset (FPA-FOD) and for Canada from the Canadian National Fire Database (CNFDB). We compared reference data to the original, unfiltered ignitions, as well as seven filtering strategies, including only ignitions that grew at least once before merging (“growth filter”), initial ignitions, ignitions of a minimum size (0.5 and 1 km^2^), and combinations of the four strategies. To identify the best filtering strategy, we created a confusion matrix of correctly and incorrectly identified single- and multi-ignition fires and computed omission and commission errors, as well as the overall accuracy and Cohen’s Kappa value (table S9). We also computed the average difference in the number of ignitions between datasets for all fires and fire complexes, as well as the fraction of fires with an identical number of ignitions in both datasets.

For California, FPA-FOD data were matched with FEDS based on FRAP fire names. Fires within FPA-FOD labeled fire complexes were aggregated for this comparison. The highest accuracy was achieved by applying a combination of growth and initial ignition filters, with initial ignitions considered as those that start within 5 days of the earliest ignition in the multi-ignition fire. This initial ignition threshold was chosen in line with the maximum temporal threshold used in the FEDS fire tracking. Table S9A shows the confusion matrix for the unfiltered and filtered versions of FEDS, compared to FPA-FOD. FPA-FOD was filtered to only include ignitions larger than 100 acres (fire size classes D to G) to approximately match the detection abilities of FEDS. It should be noted that FPA-FOD lists several known multi-ignition fires in California, such as the CZU Lightning Complex and SCU Lightning Complex in 2020, as single-ignition fires, and therefore, the actual commission error is likely lower than reported. We further conducted a visual examination of the fire progression for the remaining multi-ignition fires that did not contain multiple ignitions according to FPA-FOD. We removed two multi-ignition fires that contained artifact ignitions (often in fast-moving fuels, for example, the Sand fire in 2016). This strategy served to reduce the commission error further and create a high-confidence set of multi-ignition fires for the assessment of fire management costs and impacts using data from ICS-209 reports. The relatively large omission error in table S9 indicates that actual costs and damages from these fires may be larger than reported here. Figure S1 shows an example of the FEDS-derived ignitions compared to official records from FPA-FOD and the National Interagency Fire Center Operational Data Archive fire progression data for the August Complex fire (2020). Table S1 reports all multi-ignition fires in California that were identified through this process.

For Canada, we matched ABFA fires with CNFDB fire locations based on spatial and temporal overlap. Fires in Arctic-boreal regions can be ignited and perpetuated through several consecutive thunderstorms, a phenomenon that was not observed in California in our observational record. We therefore used both the start date (REP_DATE attribute in CNFDB) and the end date of the ABFA fires for the temporal matching. Since fires in Arctic-boreal regions can smolder for extended periods after ignition before being detected by the satellite, we adopted a more liberal threshold of 30 days for the initial ignition filtering, in line with the smoldering thresholds used in the ABFA. A combination of the growth filter and a 1-km^2^ size filter yielded the highest accuracies compared to the reference dataset and was therefore adopted for the entire ABFA dataset (table S9B).

We also compared the FEDS fire starts to fire starts derived from the GOES–Observed Fire Event Representation (GOFER GOES-West) product (*52*), which tracks fires at a higher temporal (hourly) but coarser spatial resolution for a set of large wildfires in California from 2019 to 2021. GOES imagery has a spatial resolution of ~2 km at the equator. We found that the higher temporal resolution of this product does not lead to a significantly elevated ignition detection efficiency, likely due to the tradeoff of lower spatial resolution (table S8).

We note that the actual number of individual fire starts is likely considerably higher than what is captured by the satellite data when considering more closely clustered fire starts that merge between satellite overpasses. For example, the FPA-FOD fire start dataset reports a total of 41 fire starts for the August Complex fire in 2020, but only 11 of these reach a size of more than 100 acres before merging. The relatively coarse temporal (12-hourly) and spatial (375 m) resolution of VIIRS satellite observations is not well suited to capture merging at fine temporal and spatial scales. Furthermore, the algorithm is not designed to capture spotting at the fire front or potential nearby ignitions caused by pyroCb-induced lightning. Such fires that start close to one another may also be underrepresented in fire inventories, particularly in remote areas. This underrepresentation of rapidly merging fires likely leads to an underestimation of ignition counts. However, the missed ignitions will primarily affect initial fire growth dynamics rather than the longer-term (multiday) fire behavior that constitutes the primary focus of our analysis, as multi-ignition fires in our dataset persist significantly longer than single-ignition fires and thus are dominated by fire dynamics following initial attack efforts. While we cannot determine the actual number of ignitions for rapidly merging events over intervals of less than 12 hours, our focus on large-scale fire interactions and resource allocation effects throughout the fire lifetime means that our key findings regarding multi-ignition fire behavior remain robust to this detection limitation.

### Multi-ignition versus complex fires

According to fire management agencies, for example, in California and Canada, a fire complex consists of multiple wildfires or incidents managed by a single Incident Management Team sharing resources and equipment. The satellite fire tracking we deployed differs from this management-perspective definition in that it aims only to include fires that have physically merged. The system also assembles fires that burn simultaneously and in close vicinity (1 to 5 km depending on the prevailing land cover) into fire complexes to avoid fragmentation of fires due to potential detection gaps in the active fire data.

In comparison with reference data from Incident Status Summary (ICS-209) reports and the FPA-FOD the California FEDS version returned a smaller number of complex fires for all years except 2020 (table S10). This omission was primarily caused by fires that burned in a common management area but did not physically merge or come close enough for merging to become possible or multiple fires that started so close to each other that merging happened too fast to be reliably picked up by the satellite data.

### Fire cause attribution

We used a set of reference fire databases to assess whether fires were caused by lightning or anthropogenic sources. For California, we used the fire cause attribution recorded in FRAP fire perimeters, supplemented with data from ICS-209 reports when a cause was reported as unknown.

For Alaska, we used the AWFM Fire Location database distributed by the Alaska Interagency Coordination Center, which collects causes reported by the responsible fire agencies. We overlaid all fire locations with corresponding fire perimeters of the same year based on spatial and temporal overlap, allowing for a 10-day uncertainty in the date. When several fire locations were associated with the same perimeter, they shared the same cause, so that no correction had to be applied. This crosswalk returned 440 matched fires (associated with 502 unique events in ABFA), out of which 369 were categorized as single-ignition and 71 as multi-ignition fires.

For Canada, we used the National Burned Area Composite (NBAC), which combines satellite imagery with fire data from Natural Resources Canada and Provincial, Territorial, and Parks Canada agencies. Fire causes in this database are reported by the responsible agency. We matched FEDS and NBAC perimeters by overlaying them and retaining only those FEDS perimeters with a detected burn date within a range of 10 days before to 10 days after the NBAC start date. This crosswalk returned a total of 2648 fires (2645 events according to NBAC), out of which 2214 were single-ignition and 334 were multi-ignition fires.

### Fire management data

We used ICS-209 Incident Status Summary reports to investigate hazards and resource strain associated with multi-ignition fires in California. We downloaded more than 900 ICS-209 reports for California fires from 2014 to 2023 from the FAMWeb Data Warehouse (https://www.wildfire.gov/application/famweb-data-warehouse). ICS-209 reports contain interagency updates of fire behavior and impacts, personnel, and suppression resources (e.g., engines, dozers, and helicopters), usually up to twice per day (morning and evening) for large fires during periods of active fire growth. Because ICS-209 reports are not retroactively revised, the data required intensive preprocessing to correct for various data entry errors, including numerical anomalies, missing data, duplicate incident numbers, delayed timing of reports, and data stored as text descriptions due to technical issues with tabular inputs. We downscaled the data to hourly time steps using a cubic interpolation to account for the irregular temporal frequency of the reports. For complex fires with multiple ICS-209 reports, we merged the relevant reports. Last, we cross-walked ICS-209 reports to FRAP by comparing incident numbers, fire names, the timing of ignition, spatial proximity to FRAP perimeters, and final fire size. We also searched the individual reports of 11 prominent complex fires between 2014 and 2021 for mentions of resource limitations and associated risks. Excerpts of such descriptions are summarized in table S4. We also used ICS-209-PLUS (*53*) data from 2012 to 2020 to assess the number of fires burning in a fire complex in comparison with FEDS- and GOFER-derived data (tables S1 and S9).

Limited data on fire impacts were also available for Alaska from AWFM. This dataset included records of threatened and damaged structures per fire. The methodology for matching AWFM with ABFA is described in the “Fire cause attribution” section above.

### Modeling of fire merging probability in California

Annual probabilities for the merging of fires in California were based on 1000 random simulations of fire locations during each calendar year from 2012 to 2023. For each year, the observed number of fire starts (*N*) was used, but locations were drawn from a fire probability map based on FRAP fire perimeters larger than 4 km^2^. The fire probability map was produced by computing the annual fraction of burned area from large fires recorded in FRAP in 5-km grid cells across California. The probability map was adjusted to consider fuel limitations from previous burning in forest and shrubland ecosystems. We used 30-m Global Land Cover Mapping and Estimation land cover data (*54*) for 2011 to assign each 5-km grid cell a dominant land cover type. We then created annual probability maps where grid cells were adjusted to zero if more than 10% of the grid cell had burned in the preceding 10 years for forests and 5 years for shrublands.

To account for differences in fire sizes between years, we used observed fire sizes to compute the probability of two fires merging. Fire sizes of complex fires were divided by the number of ignitions assuming that all ignitions contribute the same fraction of total area. The merging probability was assessed by computing all distances between fire starts for each simulation and comparing them to three thresholds based on radii derived from observed fire sizes (table S6): (1) the sum of the two largest radii of the year (maximum), (2) the average of all radii of the year (mean), and (3) the median of all radii of the year (median). Radii were computed from fire sizes assuming circular fire shapes. The maximum scenario (the probability of merging if all fires of a given year would reach the largest fire size radius of that year) thereby represents a case where the largest fires are occurring in locations where ignitions are spatially closest. The mean and median scenarios represent cases where distances between ignitions and fire sizes are not linked. When using the median and maximum observed distances between ignitions of multi-ignition fires as thresholds instead (i.e., disregarding differences in fire sizes between years), merging probabilities were 0.002 (median) and 0.024 (maximum) for all years.

We computed observed distances between ignitions in multi-ignition fires by computing pairwise distances between all ignition geometries. We then determined the shortest network that connects all ignitions and recorded all distances within that network.

### Effects of multiple ignitions on initial growth and fire duration

To assess the effect of multiple ignitions on initial growth, defined as the size on day 4 after the first ignition was detected, we selected a subset of fires with a total duration of at least 4 days. Not all complex fires consist of fires that started on the same day. Fire starts that are scattered throughout time can be provided by anthropogenic ignitions, multiple thunderstorms delivering lightning ignitions, or by differences in holdover (smoldering) times after ignition and before a fire is detected by the satellite due to land cover variations. For the fire size comparison, we therefore only designated fires as multi-ignition if they had two or more fire starts on the first day of the fire. For the duration we used the previously developed definition of a multi-ignition fire that considers all ignitions throughout the lifetime of a fire and also included fires with a duration of less than 4 days. Since data on fire sizes and duration were strongly right-skewed, we used the Mann-Whitney test to test for differences in medians.

We also compared the daily active fire line length recorded by FEDS and ABFA between single- and multi-ignition fires. We summed up active fire line lengths and fire sizes for all fire parts for multi-ignition fires. We then tested whether the relationship between fire area and fire line length differs between single-ignition and multi-ignition fires using linear models. We fit three models with log-transformed fire line length per unit burned area as the response variable: (1) a model with fire area and fire type (single versus multi-ignition) as additive effects, (2) a model including an interaction term between fire area and fire type, and (3) a baseline model with fire area only. We used ANOVA to compare these nested models and assess whether the interaction term and fire type significantly improved model fit, indicating different scaling relationships between single- and multi-ignition fires. The ANOVA revealed significant differences between the baseline model and the model including the fire type (*P* < 0.001 for both regions), as well as between the models with and without interaction term (slope value of models, *P* < 0.001 for Arctic-boreal regions, *P* = 0.02 for California).

For assessing differences in initial weather conditions between single- and multi-ignition fires, we computed daily mean VPD at ignition locations using ERA5-Land (fifth-generation European Centre for Medium-Range Weather Forecasts reanalysis) reanalysis data. We obtained daily mean temperature and dewpoint temperature from ERA5-Land and calculated saturated vapor pressure (SVP) and actual vapor pressure (AVP) using the Tetens equation

$$\text{SVP}=6.1078\times e^{\frac{17.2694\times \left(T-273.16\right)}{\left(T-35.86\right)}}\;\left(\text{hPa}\right)$$

where *T* is the surface air temperature in kelvin. AVP is calculated using the same equation as SVP but with temperature *T* replaced by dewpoint temperature. VPD was then computed as the difference between SVP and AVP. ERA5-Land products are available at hourly temporal resolution with a native spatial resolution of ~9 km. We extracted VPD values at the location and timing of initial ignitions and computed the mean VPD over the 4 days following the time of ignition. We tested for significant differences in VPD between single-ignition and multi-ignition fires using a two-sample *t* test.

### PyroCb inventory

All pyroCb information was obtained from a global pyroCb inventory described in detail by Peterson *et al.* (*37*), which builds from an earlier version of the inventory for 2013–2021 used in (*36*). This dataset is based in part on a growing community effort to inventory all observed pyroCb activity worldwide, called The Worldwide PyroCb Information Exchange (https://groups.io/g/pyrocb), which requires constant attention to fires and pyroCb activity in all regions worldwide. The inventory also leverages a previously developed automatic pyroCb detection algorithm applied to geostationary weather satellite observations (*30*, *35*). All entries in the inventory are listed at the pyroCb “event” level, defined as an individual pyroCb pulse or chain of several pulses (and resulting smoke injections) linked to a specific fire or segment of a large fire front (*55*).

The location of individual pyroCb events provided in the inventory can be displaced from the fire perimeters by several kilometers. For California, the pyroCb inventory contains a reference to the FRAP fire names. Since FEDS data were already cross-walked with FRAP, matching was based on fire names instead of a spatiotemporal overlay analysis. The recorded pyroCbs that had a corresponding FEDS fire in California included the following: 2021 Dixie (seven pyroCb events), 2018 Carr (two events), 2018 Cranston (two events), 2021 KNP Complex (two events), 2018 Delta (one event), 2021 Antelope (one event), 2021 Beckwourth Complex (one event), 2021 Lava (one event), 2021 McFarland (one event), and 2022 Mosquito (one event).

For Canada and Russia, we assigned each pyroCb event to the closest fire within a 20-km radius that burned at the same time. Using this technique, we linked 107 of the 164 observed pyroCb events with a fire. For the 73 pyroCb events associated with multi-ignition fires in this dataset, we compared the date of pyroCbs to the merging date of individual fires to assess whether merging could trigger pyroCb events. We identified a pyroCb as associated with merging if a merging event happened between 3 days before and 1 day after a pyroCb development.

### PyroCb simulation data

We chose the 2021 Dixie fire in California to analyze surface winds during a pyroCb event. While the Dixie fire was a single-ignition fire, it displayed two distinct fire fronts at the time when it developed a pyroCb on 9th August, allowing for the observation of potential wind patterns that could enforce fire interactions and impede firefighting operations.

The wildfire simulation conducted for this study used a global multiscale wildfire simulation framework integrated into the Energy Exascale Earth System Model (E3SM) (*56*). Key enhancements over E3SM version 2 (*57*) included the California Regionally Refined Mesh at a convection-permitting 3-km resolution (*58*), a one-dimensional plume-rise parameterization (*59*), a fire-induced vertical water vapor transport scheme, and surface wildfire sensible heat flux representation. The deep convection scheme was turned off for all grids, and large-scale dynamics were constrained in coarser resolution areas using nudging for horizontal winds, temperature, and specific humidity. The dynamic core’s time step was reduced to 9.375 s to accommodate the finer resolution, and the simulation used Cloud Layers Unified By Binormals (*60*) for turbulence and the cloud microphysics scheme described by Gettelman and Morrison (MG2) (*61*) for cloud microphysics. The Dixie Fire simulation covered 8 to 14 August 2021 (2021/08/08 00 UTC-2021/08/14 00 UTC) and used ERA5-based atmospheric initial conditions merged with prespun aerosol fields from prior output, removing the need for additional spin-up. In both cases, the free-running domain for nudging was centered at (37.2°N, 119.4°W) with a 17° × 17° extent. Nudging was applied using the product of two vertical Heaviside window functions—one for the lower and one for the upper atmosphere—scaled by a default timescale, yielding 6 hours below 100 hPa and 50 hours above. This weaker stratospheric nudging allowed two-way interactions between wildfire plumes and the large-scale circulation. Two experiments were conducted: a “Fire” run incorporating high-resolution (500-m, hourly) Fire Radiative Power data specific to the Dixie Fire and a “NoFire” run excluding fire emissions. Inside the California domain, the model atmosphere was freely evolving, while outside the domain, nudging was applied to constrain large-scale circulation. The simulated results successfully reproduced key pyroCb features, including cloud height, spatiotemporal evolution, and convective intensity. For more details, please see (*56*). The surface wind differences during peak pyroCb activity demonstrate the critical role of fire processes in shaping regional atmospheric dynamics (fig. S4).
