# Supplementary material for: Multi-ignition fire complexes drive extreme fire years and impacts
Source: Sci Adv. 2026 Jan 2;12(1):eadx6477. doi: 10.1126/sciadv.adx6477 (PMC12758541; doi:10.1126/sciadv.adx6477)
Supplement: Supplementary file 1 — Figs. S1 to S5 Tables S1 to S10 [file sciadv.adx6477_sm.pdf]

Science Advances
AAAS

Supplementary Materials for

Multi-ignition fire complexes drive extreme fire years and impacts

Rebecca C. Scholten *et al.*

Corresponding author: Rebecca C. Scholten, rebecca.scholten@uci.edu

Sci. Adv. **12**, eadx6477 (2026)
DOI: 10.1126/sciadv.adx6477

This PDF file includes:

Figs. S1 to S5
Tables S1 to S10

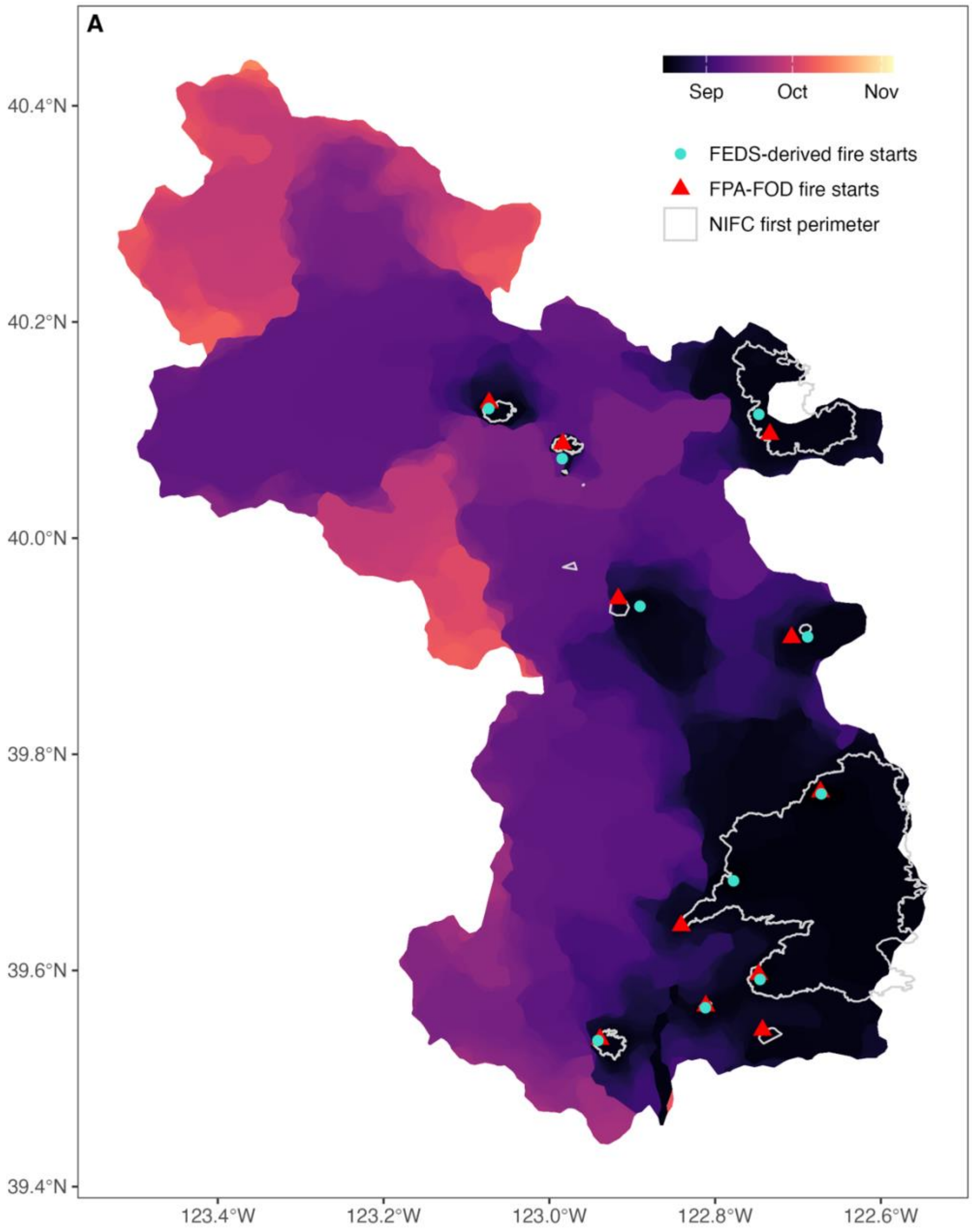

A
Sep
Oct
Nov
FEDS-derived fire starts
FPA-FOD fire starts
NIFC first perimeter
40.4°N
40.2°N
40.0°N
39.8°N
39.6°N
39.4°N
123.4°W
123.2°W
123.0°W
122.8°W
122.6°W

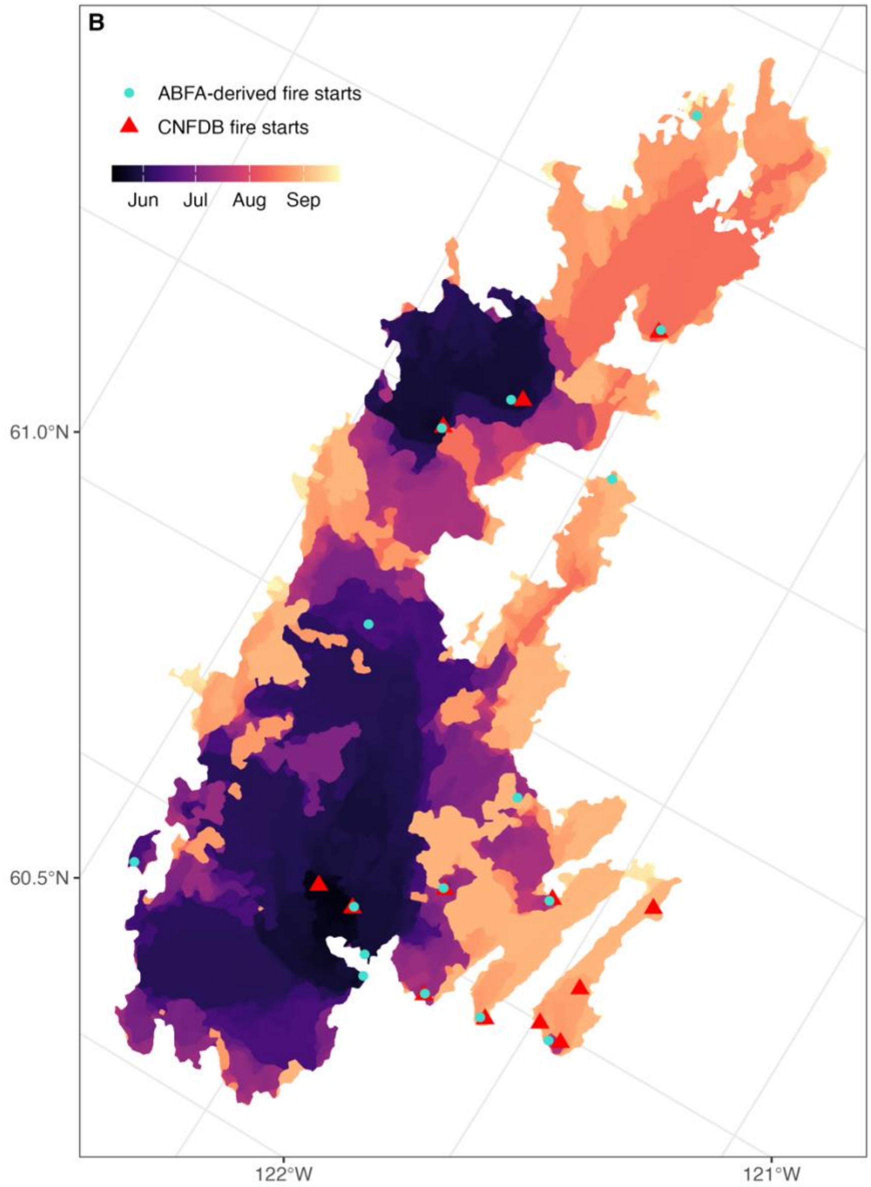

Fig. S1.

12-hourly perimeters and fire starts from FEDS/ABFA (blue diamonds) and governmental data sources (red triangles, grey outlines) for the largest fires observed in California and Canada in 2012–2023. Panel **A** shows the 2020 August Complex Fire in California. Panel **B** shows a wildfire in the Northwest Territories, Canada, in 2023. Filtering was applied to retain only true fire starts and remove artifacts caused by missing detections, as well as spot fires. For California, fire starts were filtered by start date and individual growth before merging. For the Arctic-boreal we used a minimum size and individual growth before merging threshold. The filtered FEDS/ABFA fire starts agree well with reference fire start data for both regions. NIFC: National Interagency Fire Center Incident Feature Service daily fire polygons. Fire perimeters were compiled from: August 19 at 10:24 pm (southern half of the complex), August 20 at 10:06 am (northern fire areas), and August 25 and 28 aerial surveys (small fires in northern/central areas). FPA-FOD: Fire Program Analysis Fire Occurrence Dataset (57); only fires larger than 100 acres were included. CNFDB: Canadian National Fire Database fire locations.

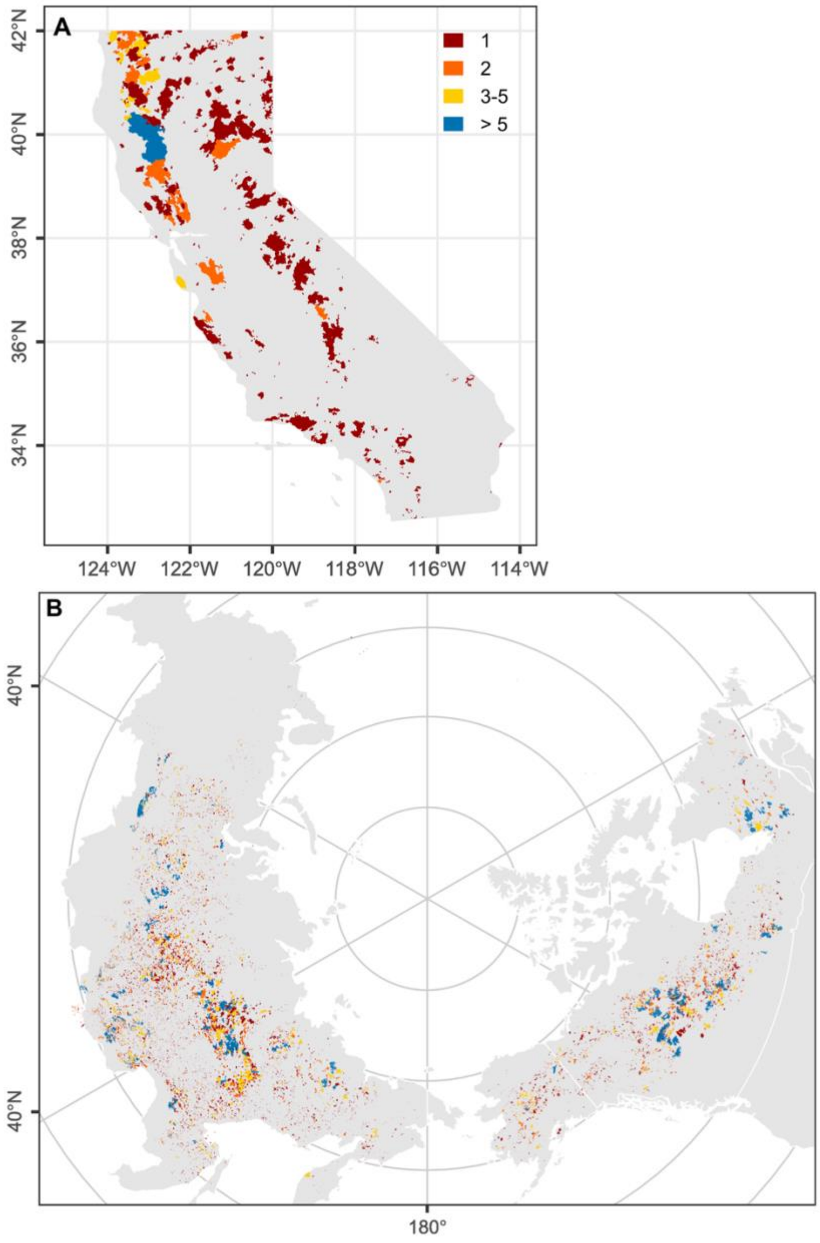

Fig. S2.

The location of single- and multi-ignition fires in California (**A**) and the Arctic-boreal domain (**B**) during 2012-2023. Fire perimeters are colored by the number of ignitions.

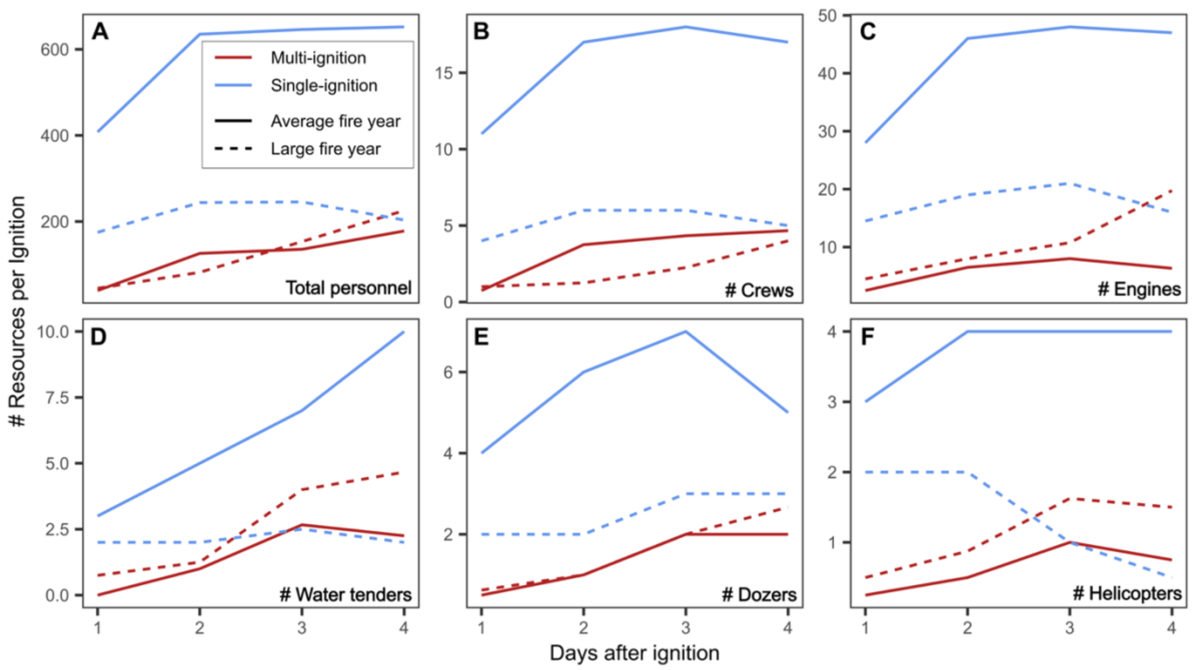

Fig. S3.

Median resource distribution per ignition during the first 4 days after the start of multi-ignition (red, n=28) and single-ignition fires (blue, n=243) in California. **A**: Total personnel; **B**: Crews; **C**: Engines; **D**; Water tenders; **E**: Bulldozers; **F**: Helicopters. Solid lines: average fire seasons (2014-2019 & 2022). Dashed lines: large fire seasons (2020 and 2021, N_{multi}=11, N_{single}=70). All median, interquartile ranges and p-values for differences between single- and multi-ignition resource distributions are shown in table S7.

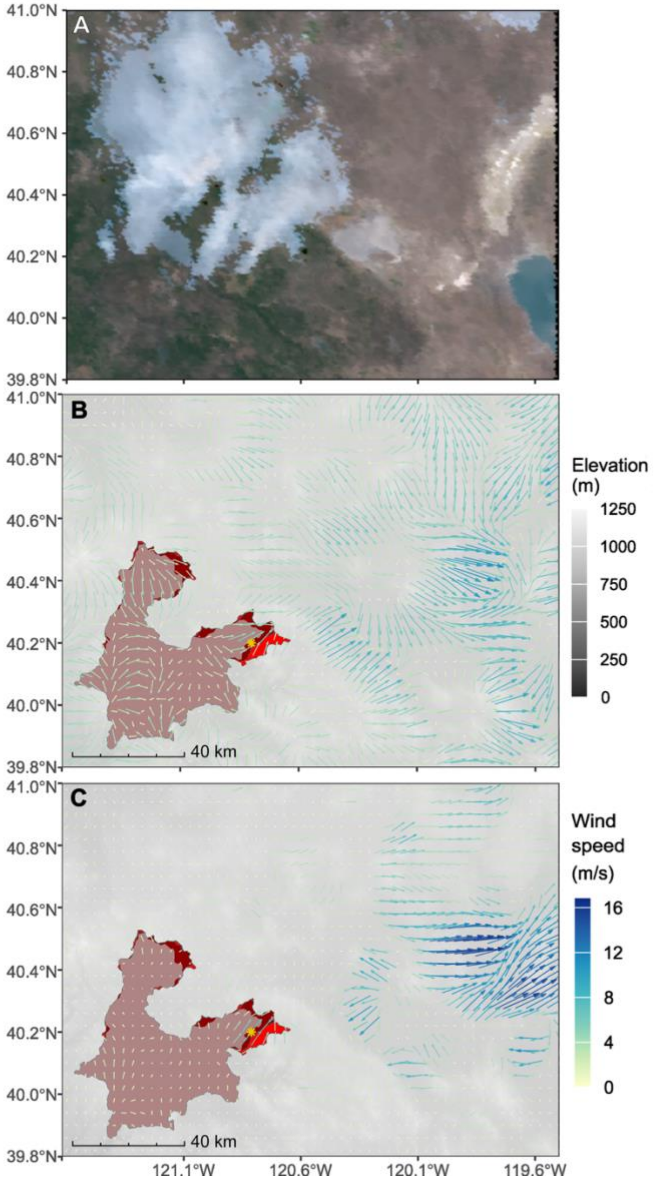

Fig. S4.

Updrafts from pyroCb development between two fire fronts can cause strong, variable surface wind fields that can impede firefighting operations and drive extreme spread. **A**, VIIRS satellite image of the Dixie fire in California on August 9, 2020, preceding a pyroCb event that developed later that day. **B**, 10-m wind speed and direction (arrows) derived from a wildfire simulation of the Dixie pyroCb at 6am UTC August 9 (see methods). **C**, The wind vectors here represent the difference between a simulation with fire emissions and sensible heat fluxes and a simulation without fire. This difference isolates the influence of the fire on near-surface winds. Yellow asterisk: pyroCb location at 6:20am UTC according to the pyroCb inventory. FEDS fire perimeters are shown for August 9, 12pm (dark red) and August 10, 12am (bright red). The gray shaded area of the fire perimeter burned prior to August 9. The background image in panel B shows the LANDFIRE 2020 Elevation product.

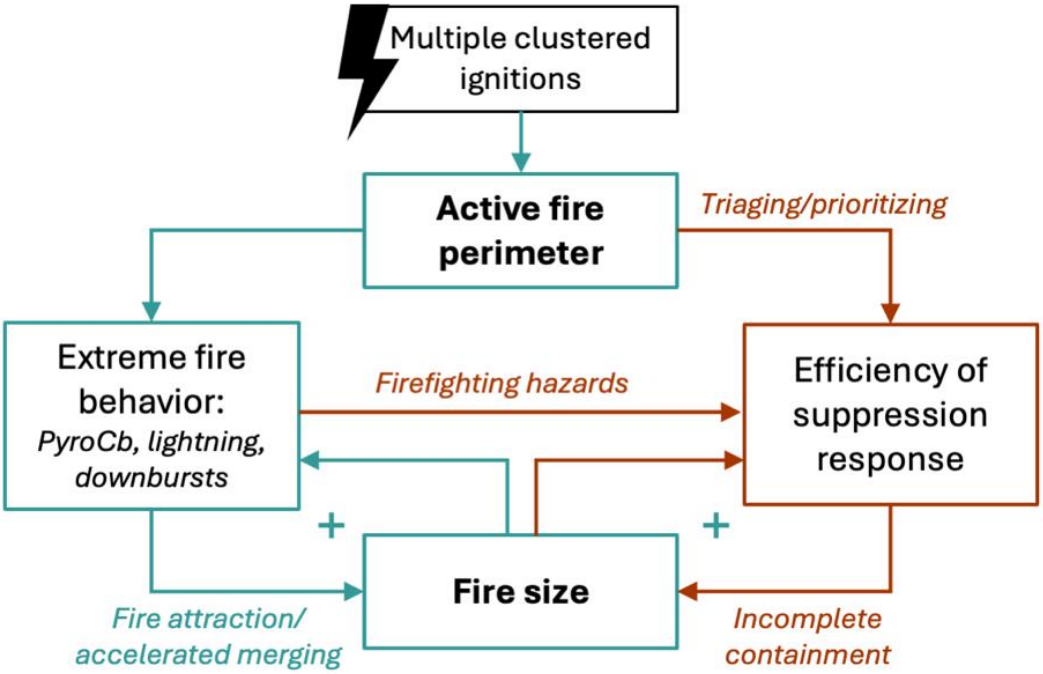

Fig. S5.

Physical and fire management feedbacks induced by multi-ignition fires lead to positive feedback loops, increasing the probability of fires becoming large and destructive. Green arrows represent positive links and brown arrows represent negative relationships. Box colors show the net effect (positive/negative) of multi-ignition fires on fire size compared to single ignitions.

Table S1.

Multi-ignition fires in California 2012-2023. The table lists the official name of the fire complex and associated fires from the Fire and Resource Assessment Program (FRAP), the number of large fire starts from FPA-FOD and ICS-209 reports (only fires that grow larger than 100 acres are included) and from FEDS, and the fire cause according to FRAP. Data from ICS reports were manually extracted from reports for 2021-2023.

Year	Complex	Fire(s)	FPA-FOD	ICS-209	FEDS	Cause
**2012**	Fort Complex	Goff	3	4	3	Lightning
**2013**	Forks Complex	Butler, Salmon Complex	3	7	2	Human
**2014**	Happy Camp Complex	Frying Pan	4	3	3	Lightning
	Basilone Complex	Pulgas	3	3	2	
**2015**	Fork Complex	Shiell, Rail, Peak	7	10	3	Lightning
	Gasquet Complex	Peak	4	7	2	Lightning
	Mad River Complex	Lassics, Gobbler, Pickett, Pine 1-44	3	4	4	Lightning
	Route Complex	Johnson	5	8	3	Lightning
**2017**	Eclipse Complex	Oak, Ukonom	7	14	2	Lightning
		Cedar			2	
	Salmon August Complex	Wallow	4	2	2	Lightning
	Southern LNU Complex	Nuns	3	2	2	Unknown
	Helena/Fork		2	-	2	Lightning/ Misc
	Modoc July Complex	Steele, Lake, Rimrock	6	3	2	Lightning
**2018**	Mendocino Complex	Ranch, River	2	2	2	Human
**2020**	August Complex		11	4	10	Lightning
	CZU Aug Lightning Complex		1	1	4	Lightning
	LNU Lightning Complex	Hennessey	6	1	2	Lightning
	North Complex		3	2	2	Lightning
	Red Salmon Complex		2	1	2	Lightning
	River		1	-	2	Lightning
	SCU Lightning Complex		1	3	2	Lightning
	Slater/Devil		2	2	2	Unknown
		1-12	-	-	2	Unknown
**2021**	River Complex		-	3	3	Lightning
	KNP Complex		-	2	2	Lightning
**2022**	2022 SRF Lightning Complex	Ammon		8	3	Lightning
**2023**	Smith River Complex	Kelly, Hurdy Gurdy, Corral	-	8	5	Lightning
	2023 SRF Lightning Complex	Mosquito	-	8	2<br>5	Lightning

Table S2.

Median, interquartile range (IQR), and Mann-Whitney p-value statistics of burned area per ignition, burned area per ignition on day four, duration, and distance between ignitions for single- and multi-ignition fires.

Variable	Group	Region	Median	IQR	N	p-value
**Burned area per ignition (km^2)**	Single-ignition	California	22.0	8.9 – 80.0	377	< 0.001
	Multi-ignition		103.2	51.0 – 267.8	30	
	Single-ignition	Arctic-boreal	14.0	7.1 – 34.2	14,305	< 0.001
	Multi-ignition		44.4	17.2 – 117.1	2,161	
**Burned area per ignition on day 4 (km^2)**	Single-ignition	California	24.8	6.65 – 92.5	180	0.62
	Multi-ignition		20.9	6.60 – 80.7	29	
	Single-ignition	Arctic-boreal	5.7	2.2 – 13.0	12171	< 0.001
	Multi-ignition		8.4	3.4 – 19.4	1205	
**Duration (days)**	Single-ignition	California	3.5	1.0 – 10.0	377	< 0.001
	Multi-ignition		26.8	14.5 – 36.8	30	
	Single-ignition	Arctic-boreal	10.0	4.0 – 21.5	14,305	< 0.001
	Multi-ignition		28.0	16.0 – 48.0	2,161	
**Distance between ignitions (km)**	Multi-ignition	California	9.5	6.4 – 13.4	54	
		Arctic-boreal	8.2	5.1 – 13.2	4178	

Table S3.

Average burned area (BA, km^2) from single- and multi-ignition fires in average and large fire years and coefficient of variation for California and Arctic-boreal regions in 2012–2023. Large fire years were identified as years with a burned area larger than the mean + one standard deviation; for California: 2020, 2021; Alaska: 2015, 2019, 2022; Canada: 2023; Russia: 2012, 2021. The coefficient of variation was computed from the mean and standard deviation of the burned area time series.

	**Average fire years**			**Large fire years**			**Coefficient of variation**		
**Region**	Mean BA (single)	Mean BA (multi)	% BA (multi)	Mean BA (single)	Mean BA (multi)	% BA (multi)	Single-ignition	Multi-ignition	All fires
**California**	2,212	578	20.7	8,297	5,951	41.8	0.84	1.77	1.06
**Alaska**	1,064	465	30.4	5,986	7,140	54.4	1.03	1.51	1.25
**Canada**	9,013	12,116	57.3	36,208	116,614	76.3	0.79	1.51	1.24
**Russia**	30,313	34,003	52.9	46,321	92,056	66.5	0.35	0.60	0.47

Table S4.

Fire management resources and loss and damage due to multi-ignition (N=28) and single-ignition fires (N=290). For the comparison per ignition all resources and damages were divided by the number of ignitions for each fire. Following ICS-209 protocol, affected civilians and responders include those who are: injured, ill, or deceased; trapped or missing; sheltering in place, quarantined, requiring or having received immunizations; and evacuated or in temporary shelters (civilians only).

		**Multi-ign. fires (total)**		**Single-ign. fires**		**Multi-ign. fires (per ignition)**		**Mann-Whitney test p-value**	
		Median	*IQR*	*Median*	*IQR*	*Median*	*IQR*	*Total*	*Per ignition*
**Resources**	Incident cost (million $)	50.7	32.8 – 97.0	11.5	3.9 – 34.8	23.0	13.5 – 34.2	< 0.001	0.01
	Total personnel (number of people)	1,766.0	1,227.5 – 2,598.3	971.0	542.3 – 1,899.5	690.6	471.4 – 1,024.1	< 0.001	0.06
	# Engines	139.5	108.8 – 253.3	90.0	30.3 – 176.8	54.2	37.5 – 86.4	< 0.001	0.29
	# Helicopters	11.5	8.0 – 21.0	9.0	4.0 – 15.0	4.8	3.4 – 8.0	< 0.001	0.04
	# Bulldozers	25.0	19.5 – 44.5	14.0	5.0 – 29.0	11.3	6.8 – 17.6	< 0.001	0.63
	# Water tenders	31.0	23.0 – 47..8	16.0	7.0 – 31.0	13.1	9.7 – 21.1	< 0.001	0.61
**Loss & Damage**	Threatened structures	1,869.5	451.5 – 13,768.8	249.0	24.0 – 1,470.3	887.8	150.5 – 3,946.2	< 0.001	0.005
	Damaged structures	2.0	0.0 – 29.0	0.0	0.0 – 3.0	0.8	0.0 – 14.5	0.003	0.02
	Destroyed structures	10.0	1.8 – 320.0	2.0	0.0 – 20.0	3.3	0.6 – 119.0	0.004	0.04
	Affected civilians	981.0	194.3 – 17,631.3	150.0	0.0 – 1,463.5	366.3	78.9 – 8,610.5	0.002	0.04
	Evacuated civilians	943.5	186.8 – 17,200.0	117.5	0.0 – 1,409.0	360.7	78.9 – 8,600.0	0.001	0.03
	Affected responders	12.0	3.0 – 28.5	2.0	0.0 – 6.0	4.0	1.4 – 14.3	< 0.001	0.04

Table S5.

Selected excerpts of ICS-209 reports for prominent multi-ignition fires in California illustrating resource limitations and associated risks.

Year	Fire name	Start date	Report date	Excerpts
2014	July Complex		08/05 – 08/06	The zero percent containment in block 8 is due to continuing indirect line construction due to **limited suppression resources**, steep/rugged terrain and aggressive fire behavior.
2014	Happy Camp Complex	08/11	08/16	Management opportunities on the Frying Pan are being reevaluated with the possibility of Frying Pan and Falkstien **growing together**. Additional crew resources are needed to support projected opportunities. The Falkstein fire is estimated at 350 ac and remains **unstaffed**.
			08/18	A strong inversion held until mid-afternoon when it was broken up by thunderstorms developing of the Complex. Lightning strikes forced crews to disengage and/or shelter from the lightning<br>Hunters in the area are in danger of being trapped by both the Frying Pan and Falkstein fires.
			08/29	Steep terrain, **long travel times around the fire** and poor air quality continue to slow and hamper fire suppression efforts
2015	Fork Complex	07/30	07/31	[…] limited availability of ground and air resources<br>Several other large fires in the area continue to draw resources.
			07/31– 08/02	Fatigue management will still be a priority until sufficient resources are assigned.
			07/31– 08/06	The amount of fires has far exceeded the initial attack capabilities and the majority of these fires are currently unstaffed. An Initial Attack Area can only be identified once adequate resources arrive.
			08/01– 08/15	Reduce firefighter exposure by only engaging fires with the highest probability of success.
			08/03– 08/15	The current helicopter medivac alternative does not provide for a timely response (>45 min) and transportation of injured firefighters.
			08/03	Aviation assets are in jeopardy of being shut down due to the lack of available aviation frequencies and the inability to establish TFRs.<br>Due to a complex airspace coordination of multiple incidents an ATGS Platform is required to prevent shutdown of aviation assets.
			08/03– 08/12	Given the current limited number of resources assigned and the fires growth/terrain/complexity will drastically extend containment objectives and further force evacuations. […] It is unlikely to achieve incident management targets with current resources and constraints.
2017	Orleans Complex	07/25	08/29 – 09/25	All aviation **resources are being shared** [with the Salmon August Complex, Eclipse Complex, and for Initial Attack on the SRF and KNF].
		07/25	09/02 – 09/06	Critical orders to support SOP and structure protection plan **remain unfilled**. Minimum critical orders to keep current resource levels in days to come remain unfilled. Resources continue to **demob with no backfill**.
		07/25	09/03	The Wallow fire of the Salmon August complex is approximately 5 miles from the Haypress Fire and its **impact of combining** with the Haypress fire and associated increased uncontained fire perimeter (along with the threat to Salmon River Road and nearby communities) will **require additional resources** beyond what is listed here in order to adequately mitigate the threat to life and property. […]<br>With **limited resources**, snag hazards, and difficult steep access, successful containment was not probable.

	Eclipse Complex	08/15	09/07	Several Divisions are **downstaffed to patrol status** due to **lack of Overhead and Crews**
			09/07	Priorities for the **limited resources** include: […]<br>Division Z and F will be **unstaffed** but patrolled due to **limited resources**.<br>Due to **limited resource availability**, the Cedar Fire will be unstaffed, but patrolled for the next operational period.
			09/07	**Limited resources**, UTF orders, and crews timing out have caused several divisions to be **down-staffed or forced into monitor status due to lack of engines and crews** to support the operations. Steep and rocky terrain with significant dead and down fuels, snags, and long travel times on steep mountain roads create a significant challenge for suppression operations with the **limited resources**.<br>Due to the **lack of engines and crews** divisions have been forced into monitor status or down-staffed. […] UTF orders, **limited and timing out resources** will make it increasingly difficult to support the incident priorities, strategy and tactics while the threats to the areas and values at risk remain valid.
			09/07	**Lack of HEQB's** will prohibit the use of heavy equipment on vital areas of the fires, this includes direct suppression actions, contingency and suppression repair.
2018	Mendocino Complex	07/27	07/28 – 07/30	A **lack of suppression and overhead resources** will challenge todays operational plan.
2020	CZU AUG lightning	8/16	08/19	A FMAG was submitted last night (8/18). The fires have begun to **merge** and have made significant runs overnight destroying multiple structures and heading into the San Lorenzo Valley including Boulder Creek, forcing large scale evacuations of over 22,000 people.
			08/20	Statewide **drawdown of resources** has created challenges to supporting the incident priorities. Crews are focusing on life safety threats **until sufficient equipment arrives** to initiate perimeter control and indirect actions.
			08/22 – 08/27	Active fire and a **lack of resources** to mop up interior burn is slowing the progress for DINS teams.
			08/24 – 08/28	Heavy fuels and steep rugged terrain are keeping progress slow with the **limited resources available.**
	August complex	08/17	08/18	(explain major problems and concerns) HEAT, WINDS, **RESOURCE NEEDS**
			08/19	This complex includes 32 fires with more fires expected to be detected as fuels continue to dry out. Complexities include: **limited resources** and logistical difficulties managing **multiple fires** in steep, rugged terrain with limited connectivity and long distances.
			08/21	Complexities include: **limited resources** and logistical difficulties managing **multiple fires** in steep, rugged terrain with limited connectivity and long distances. Extreme fire behavior including **group torching and spotting** impedes control efforts. Aircraft are being utilized to support firing operations, but **aircraft are limited**. [..] We have had **difficulty assessing fire size and growth** due to multiple UTF of infrared operations and the size of the complex leading to **insufficient IR time**.
			08/24<br>08/25	The **number, type, and pace of incoming resources are completely inadequate** given the size of the complex, fire behavior, **logistical difficulties managing multiple fires** in steep, rugged terrain with limited connectivity, numerous values at risk, and long distances necessitating 3 hour drives between fires. Operations have been **limited by lack of resources**. Active fire behavior including group torching and spotting on multiple adjacent fires impedes control efforts. Aircraft are being utilized to support firing operations, but **aircraft are limited**.
	LNU LC	08/17	08/19 – 01/09	**Very limited resources** assigned to the incident.
			08/19	A **lack of critical resources** in the State has forced the Initial Attack resources to be **double shifted**.

			08/20 – 08/28	Very limited resources assigned to the incident has caused resources to work **longer than normal shifts altering the work to rest cycle.**
			08/20 – 09/01	The fires will continue to be staffed with the limited resources, making structure defense and perimeter control difficult.
2021	River Complex	07/30	08/01	Continue to staff fires with resources available. Continue to access and get size ups of current fires. Fully suppress fires when staffed. Continue to **prioritize** fires focusing on fires with higher growth potential and values at risk.
			08/01 – 08/02	**Multiple fires** and being able to staff with **limited resources**.
			08/02 – 08/03	We are engaged in point protection at this point due to fire activity and limited resources.
			09/08	Current **resources are in-sufficient** to complete the **multiple burns** we have underway. (at time of merging)
			09/01 – 09/28	The current organization is already **understaffed** and with time out dates will become worse and span of control will become **in-sufficient**.
2021	KNP Complex	09/11	09/12 – 09/14	Currently have **insufficient resources of all types** (crews, engines, overhead) to execute the plan to control the fire and protect values at risk.

Table S6.

Annual probabilities for merging of fires in California. The observed column represents the probability that any two large fires will merge based on the satellite observations. The simulated probability is derived from a model analysis we undertook to explore the probability of two large fire starts merging due to random chance from ignition locations that were randomly distributed over the landscape. In the simulation, for each year, the observed number of fire starts (N) was used, but locations were drawn from a fire probability map we created using FRAP fire perimeters larger than 1000 acres. Merging probability was assessed by computing all distances between fire starts for each simulation and comparing them to three thresholds based on radii derived from observed fire sizes: (1) the sum of the two largest radii of the year (maximum), (2) the average of all radii of the year (mean) and (3) the median of all radii of the year (median). Radii were computed from fire sizes assuming circular fire shapes. Fire sizes of multi-ignition fires were divided by the number of ignitions.

**Year**	**N**	$N_{multi\text{-}ignition}$	**Observed probability of merging**	**Simulated probability**		
				Maximum	Mean	Median
2012	34	3	0.088	0.012	0.001	0.001
2013	28	2	0.071	0.012	0.002	0.001
2014	35	5	0.143	0.008	0.001	0.001
2015	42	12	0.286	0.012	0.002	0.001
2016	35	0	0.000	0.009	0.001	0.001
2017	66	12	0.182	0.016	0.002	0.001
2018	31	2	0.065	0.023	0.003	0.001
2019	22	0	0.000	0.008	0.001	0.001
2020	84	28	0.333	0.025	0.003	0.001
2021	37	5	0.135	0.039	0.004	0.002
2022	18	3	0.167	0.007	0.002	0.001
2023	29	12	0.414	0.003	0.001	0.001
**Mean**	38.4	7.0	0.157	0.015	0.002	0.001

Table S7.

Median and interquartile range (IQR) of resources per ignition during the first four days after the start of multi-ignition and single-ignition fires for large (2020 & 2021, N_{multi}=11, N_{single}=70) and average fire years (2014–2019, 2022, N_{single}=17, N_{single}=173). P-values are based on Mann-Whitney tests.

	Resource	Days after ignition	**Single-ignition fires**		**Multi-ignition fires**		**p-value**
			Median	IQR	Median	IQR	
**Large fire years (2020 & 2021)**	Total personnel	1	175.0	55.0–443.0	44.8	14.6–86.1	0.01
		2	244.0	107.8–583.0	82.0	32.0–132.1	0.02
		3	245.5	133.3–736.5	153.4	80.4–257.9	0.09
		4	203.5	20.8–773.8	225.5	168.6–321.4	0.85
	# Crews	1	4.0	1.0–10.3	1.0	0.6–1.7	0.01
		2	6.0	2.8–12.3	1.3	0.8–2.8	0.004
		3	6.0	2.8–15.0	2.3	1.2–4.0	0.01
		4	5.0	0.0–14.5	4.0	3.5–4.8	0.59
	# Engines	1	14.5	2.0–35.0	4.5	0.5–8.6	0.06
		2	19.0	4.0–50.3	8.0	1.8–18.3	0.15
		3	21.0	4.0–59.0	10.8	4.0–28.7	0.28
		4	16.0	0.5–54.8	19.8	10.7–37.8	0.69
	# Water tenders	1	2.0	0.0–6.0	0.8	0.0–2.0	0.18
		2	2.0	0.0–6.3	1.3	0.1–4.4	0.60
		3	2.5	0.8–8.0	4.0	0.6–5.4	0.77
		4	2.0	0.0–9.3	4.7	1.5–7.8	0.74
	# Dozers	1	2.0	0.0–6.3	0.6	0.1–2.8	0.22
		2	2.0	0.0–10.0	1.0	0.4–5.4	0.59
		3	3.0	0.0–10..3	2.0	0.5–6.9	0.83
		4	3.0	0.0–8.8	2.7	1.4–6.6	0.86
	# Helicopters	1	2.0	0.0–4.0	0.5	0.0–1.4	0.09
		2	2.0	0.0–5.0	0.9	0.0–1.5	0.16
		3	1.0	0.0–6.0	1.6	0.6–2.4	0.99
		4	0.5	0.0–4.8	1.5	0.9–3.5	0.47
**Average fire years (2014-2019, 2022)**	Total personnel	1	408.0	196.0–702.0	39.8	4.5–120.0	< 0.001
		2	635.0	272.0–1058.0	126.0	23.5–182.5	< 0.001
		3	646.0	285.0–1467.0	135.5	83.5–266.5	< 0.001
		4	652.0	307.3–1467.8	178.0	53.5–224.3	0.004
	# Crews	1	11.0	4.0–20.0	0.8	0.0–4.0	< 0.001
		2	17.0	7.0–28.0	3.8	0.7–5.7	< 0.001
		3	18.0	7.0–35.0	4.3	1.7–5.8	< 0.001
		4	17.0	5.8–32.5	4.7	1.3–5.4	0.002
	# Engines	1	28.0	11.0–68.0	2.5	0.0–11.5	< 0.001
		2	46.0	15.0–101.0	6.5	0.5–19.0	< 0.001
		3	48.0	17.0–119.0	8.0	3.0–30.5	< 0.001
		4	47.0	11.0–113.5	6.3	3.0–19.0	0.01
	# Water tenders	1	3.0	0.0–7.0	0.0	0.0–2.0	< 0.001
		2	5.0	2.0–12.0	1.0	0.0–3.5	< 0.001
		3	7.0	2.0–17.0	2.7	0.0–5.3	0.002
		4	10.0	2.0–19.3	2.3	0.0–5.3	0.02
	# Dozers	1	4.0	1.0–10.0	0.5	0.0–1.3	< 0.001
		2	6.0	2.0–13.0	1.0	0.0–2.8	< 0.001
		3	7.0	2.0–13.0	2.0	0.3–4.5	0.005
		4	5.0	0.8–16.0	2.0	1.3–3.0	0.12
	# Helicopters	1	3.0	0.0–6.0	0.3	0.0–1.5	0.0003
		2	4.0	1.0–8.0	0.5	0.0–1.8	< 0.001
		3	4.0	1.0–9.0	1.0	0.0–2.0	0.002
		4	4.0	0.0–9.0	0.8	0.3–1.4	0.04

Table S8.

Comparison of the number of fire starts for 21 large fires that burned over 50 000 acres (202 km^2) in California from 2019 to 2021 from the GOES-Observed Fire Event Representation (GOFER) based on GOES-West, the FEDS-derived approach used here, and FPA-FOD.

**Fire name**	**Year**	**# Ignitions**		
		**GOFER**	**FEDS (filtered)**	**FPA-FOD (large)**
Kincade	2019	1	1	1
Walker	2019	1	1	1
August Complex	2020	8	10	11
Bobcat	2020	1	1	1
Creek	2020	1	1	2
CZU Lightning Complex	2020	3	4	1
Dolan	2020	1	1	1
Glass	2020	1	1	1
July Complex	2020	4	1	4
LNU Lightning Complex	2020	9	4	6
North Complex	2020	6	2	3
Red Salmon Complex	2020	2	2	2
SCU Lightning Complex	2020	4	4	1
Slater and Devil	2020	2	2	1
SQF Complex	2020	2	1	2
W-5 Cold Springs	2020	1	1	1
Zogg	2020	1	1	1
Dixie	2021	6	1	-
KNP Complex	2021	2	2	-
River Complex	2021	3	4	-
Tamarack	2021	2	1	-

Table S9.

Accuracy of multi- and single-fire identification from our approach (FEDS and ABFA-derived) and US and Canada national fire datasets. In part **A** of the Table, we compare our FEDS-derived approach with the USFS FPA-FOD database. In this analysis, we had 274 matched fires and fire complexes. In part **B** we compare our ABFA-derived approach with fire locations from the Canadian National Fire Database fire locations. In this analysis we had 2185 matched fires and fire complexes. The eight versions of FEDS/ABFA are based on different filtering strategies for ignitions: unfiltered (No Filter), ignitions that grew at least once before merging (Growth), initial ignitions only, minimum size filters (0.5 and 1 km^2) and a combination of growth and all other filters. The filtering strategy with the best accuracy metrics is highlighted in gray. *Single* and *Multi* refer to the number of matched fires with one or several fire locations in the reference dataset and FEDS/ABFA displayed as a confusion matrix. *Average diff.* refers to the mean difference in number of ignitions between both datasets (pairwise comparisons) across all matched fires. *Fraction correct* refers to the fraction of fires with a matching number of ignitions between both dataset. For California, FEDS fires were matched with Fire and Resource Assessment Program (FRAP) fire names and complex names, and fires within fire complexes were aggregated for the comparison. FPA-FOD was filtered by ignitions that grew over 100 acres (fire size classes D-G).

**A**		**FPA-FOD**		**Commission Error**	**Omission Error**	**Accuracy**		**# Ignitions per fire**	
		Single	*Multi*			**Overall**	**Kappa**	**Average diff.**	**Fraction correct**
**Unfiltered**	*Single*	208	22	0.10	0.09	0.85	0.44	-0.03	0.80
	Multi	20	24	0.45	0.48				
**Growth filter**	*Single*	217	23	0.10	0.05	0.88	0.50	-0.14	0.83
	Multi	11	23	0.32	0.50				
**Initial ignition filter**	*Single*	216	23	0.10	0.05	0.87	0.49	-0.11	0.83
	Multi	12	23	0.34	0.50				
**Fires > 0.5 km^2**	*Single*	212	24	0.10	0.07	0.85	0.44	-0.15	0.84
	Multi	16	22	0.42	0.52				
**Fires > 1 km^2**	*Single*	215	24	0.10	0.06	0.86	0.47	-0.15	0.84
	Multi	13	22	0.37	0.52				
**Growth + Initial**	*Single*	222	24	0.10	0.03	0.89	0.54	-0.18	0.85
	Multi	6	22	0.21	0.52				
**Growth + 0.5 km^2**	*Single*	218	24	0.10	0.04	0.88	0.49	-0.09	0.82
	Multi	10	22	0.31	0.52				
**Growth + 1 km^2**	*Single*	219	24	0.10	0.04	0.88	0.50	-0.12	0.83
	Multi	9	22	0.29	0.52				

B		CNFD		Commission Error	Omission Error	Accuracy		# Ignitions per fire	
		Single	*Multi*			Overall	Kappa	Average diff.	Fraction correct
**Unfiltered**	*Single*	1471	130	0.08	0.17	0.80	0.43	0.44	0.73
	Multi	311	273	0.53	0.32				
**Growth filter**	*Single*	1660	179	0.10	0.07	0.86	0.52	-0.02	0.81
	Multi	122	224	0.35	0.44				
**Initial ignition filter**	*Single*	1492	139	0.09	0.16	0.80	0.43	0.31	0.73
	Multi	290	264	0.52	0.34				
**Fires > 0.5 km^2**	*Single*	1585	163	0.09	0.11	0.84	0.47	0.17	0.77
	Multi	197	240	0.45	0.40				
**Fires > 1 km^2**	*Single*	1619	172	0.10	0.09	0.85	0.49	0.09	0.79
	Multi	163	231	0.41	0.43				
**Growth + Initial**	*Single*	1667	187	0.10	0.06	0.86	0.51	-0.07	0.81
	Multi	115	216	0.35	0.46				
**Growth + 0.5 km^2**	*Single*	1670	183	0.10	0.06	0.86	0.52	-0.04	0.81
	Multi	112	220	0.34	0.45				
**Growth + 1 km^2**	*Single*	1680	189	0.10	0.06	0.87	0.52	-0.06	0.82
	Multi	102	214	0.32	0.47				

Table S10.

Annual number of multi-ignition fires in California based on FEDS and four different reference datasets. ICS-209: Incident Status Summary reports of daily wildfire situation. FPA-FOD: Fire Program Analysis fire occurrence database. For ICS-209, complex fires are estimated as the number of fires with 'complex' in their name. For FPA-FOD, complex fires are estimated as the number of fires complexes listed as having a complex name.

Year	ICS-209	FPA-FOD	FEDS
2012	5	12	**1**
2013	2	5	**1**
2014	8	8	**2**
2015	9	9	**4**
2016	2	2	**0**
2017	11	12	**6**
2018	2	2	**1**
2019	0	0	**0**
2020	9	9	**9**
2021	4	*NA*	**2**
2022	1	*NA*	**1**
2023	7	*NA*	**3**
